# NCBench: providing an open, reproducible, transparent, adaptable, and continuous benchmark approach for DNA-sequencing-based variant calling

**DOI:** 10.12688/f1000research.140344.1

**Published:** 2023-09-11

**Authors:** Friederike Hanssen, Gisela Gabernet, Nicholas H. Smith, Christian Mertes, Avirup Guha Neogi, Leon Brandhoff, Anna Ossowski, Janine Altmueller, Kerstin Becker, Andreas Petzold, Marc Sturm, Tyll Stöcker, Sugirthan Sivalingam, Fabian Brand, Axel Schmidt, Andreas Buness, Alexander J. Probst, Susanne Motameny, Johannes Köster

**Affiliations:** 1 Quantitative Biology Center, Eberhard Karls University Tübingen, Tübingen, Germany; 2 TUM School of Computation, Information and Technology, Technical University of Munich, Munich, Germany; 3 Institute of Human Genetics, Klinikum rechts der Isar, School of Medicine, Technical University of Munich, Munich, Germany; 4 Munich Data Science Institute, Technical University of Munich, Munich, Germany; 5 Cologne Center for Genomics, University of Cologne, Cologne, Germany; 6 West German Genome Center - Cologne, University of Cologne, Cologne, Germany; 7 Max Delbrück Center for Molecular Medicine in the Helmholtz Association (MDC), Berlin, Germany; 8 Core Facility Genomics, Berlin Institute of Health at Charité - Universitätsmedizin Berlin, Berlin, Germany; 9 DRESDEN-concept Genome Center, TUD Dresden University of Technology, Dresden, Germany; 10 Institute of Medical Genetics and Applied Genomics, University Hospital Tuebingen, Tübingen, Germany; 11 Institute of Crop Science and Resource Conservation, University of Bonn, Bonn, Germany; 12 Institute of Human Genetics, Medical Faculty and University Hospital Düsseldorf, Heinrich-Heine-University Düsseldorf, Düsseldorf, Germany; 13 Institute for Genomic Statistics and Bioinformatics, Medical Faculty, University of Bonn, Bonn, Germany; 14 Institute of Human Genetics, University Hospital of Bonn, Bonn, Germany; 15 Core Unit for Bioinformatics Analysis, University Hospital Bonn, Bonn, Germany; 16 Environmental Metagenomics, Research Center One Health Ruhr, University Alliance Ruhr, Faculty of Chemistry, University of Duisburg-Essen, Essen, Germany; 17 Bioinformatics and Computational Oncology, Institute for Artificial Intelligence in Medicine (IKIM), University Medicine Essen, University of Duisburg-Essen, Essen, Germany; 18 German Cancer Consortium, Essen, Germany

**Keywords:** continuous, benchmarking, NGS, variant calling

## Abstract

We present the results of the human genomic small variant calling benchmarking initiative of the German Research Foundation (DFG) funded Next Generation Sequencing Competence Network (NGS-CN) and the German Human Genome-Phenome Archive (GHGA).

In this effort, we developed NCBench, a continuous benchmarking platform for the evaluation of small genomic variant callsets in terms of recall, precision, and false positive/negative error patterns. NCBench is implemented as a continuously re-evaluated open-source repository.

We show that it is possible to entirely rely on public free infrastructure (Github, Github Actions, Zenodo) in combination with established open-source tools. NCBench is agnostic of the used dataset and can evaluate an arbitrary number of given callsets, while reporting the results in a visual and interactive way.

We used NCBench to evaluate over 40 callsets generated by various variant calling pipelines available in the participating groups that were run on three exome datasets from different enrichment kits and at different coverages.

While all pipelines achieve high overall quality, subtle systematic differences between callers and datasets exist and are made apparent by NCBench.These insights are useful to improve existing pipelines and develop new workflows.

NCBench is meant to be open for the contribution of any given callset. Most importantly, for authors, it will enable the omission of repeated re-implementation of paper-specific variant calling benchmarks for the publication of new tools or pipelines, while readers will benefit from being able to (continuously) observe the performance of tools and pipelines at the time of reading instead of at the time of writing.

## Introduction

Genome sequencing is integral to many research and diagnostic procedures. For both pipeline and tool development, it is crucial to ensure that genomic variant calls are as accurate as possible. This can be achieved by testing tools and pipelines on datasets with a known set of true variants and correspondingly known sites where the genome is the same as the reference genome.

Several such benchmark datasets have been published. The Genome in a Bottle Consortium (GIAB) has released truth variant sets based on common calls across three variant callers on 14 different sequencing technologies and library preparation methods on a well-characterized genome (HG001 or NA12878), as well as an Ashkenazim trio (HG002-4) and a Han Chinese trio (HG005-7). ^1^^,^^2^ The Platinum variant catalog provides consensus calls of six variant calling pipelines across two different sequencing platforms on a family of four grandparents, two parents and 11 children including the NA12878 genome, allowing an extended inheritance-based validation. ^3^ In an alternative approach, Li et al. ^4^ generated a synthetic diploid from two complete hydatidiform mole (CHM) cell lines (CHM1 and CHM13), which are almost completely homozygous across the whole genome, such that the known variants in this set are phased (their haplotype of origin is known). The synthetic diploid benchmark dataset has the advantage of not relying on a consensus callset across several variant callers, which limit the benchmark set to high-confidence regions and lead to an overestimation of the true variant calling performance. Finally, the SEQC2/MAQC-IV initiative provides another extensive set of validated benchmarks, not only focussing on genomic DNA but also considering RNA-seq and single-cell sequencing. ^5^

Several publications have utilized the aforementioned gold-standard callsets to benchmark variant calling tools and pipelines. ^6^^–^^9^ However, the continuous development of variant calling tools and pipelines means that static, one-time benchmarks based on a specific pipeline or tool version can quickly become outdated.

In contrast, benchmarking platforms aim at providing a way to facilitate continuous benchmarking by pipeline and tool developers and users. Examples of such platforms are OpenEBench [ 1] and Omnibenchmark [ 2]. Both platforms run on their own dedicated computing infrastructure and utilize specialized frameworks for results reporting and dataset uploading.

In this work, we want to propose a different approach for hosting a continuous benchmark, which was developed by the human genomic small variant calling benchmarking initiative of the NGS-CN [ 3] and GHGA [ 4]. We show that it is possible to build a benchmarking platform by entirely relying on public free infrastructure, namely GitHub [ 5], GitHub Actions [ 6], and Zenodo [ 7]. Using these technologies as a basis and extending upon best practices, ^10^ we developed a comprehensive and reproducible benchmarking workflow for small genomic variants that is agnostic of the used dataset and can evaluate an arbitrary number of given callsets, while reporting the results in a visual and interactive way.

## Methods

### Datasets

We have sequenced the NA12878 sample from the genome in a bottle (GIAB) [ 8] project with two exome sequencing kits at varying average coverages. The genomic DNA from NA12878 was obtained from the NIGMS Human Genetic Cell Repository at the Coriell Institute for Medical Research. The Agilent Human All Exon V7 kit was used to yield a dataset with 182 million paired-end reads sequenced on an Illumina Nova Seq 6000 (211 bp mean insert size and 2 × 101 bp read length). We used random subsampling to derive two datasets from this that were used in the benchmarking, one with 37.5 million and one with 100 million paired-end reads. The Twist Human Comprehensive Exome (Twist Bioscience, San Francisco, CA, USA) sequencing kit was used according to the manufacturer’s protocol to generate 200 million paired-end reads on an Illumina NovaSeq 6000 (291 bp mean insert size and 2 × 101 bp read length). The raw reads of the two subsampled Agilent and Twist exome datasets are available via Zenodo. ^11^^,^^12^

### Evaluation pipeline

To analyze the quality of the callsets yielded by each pipeline on the given datasets, we have developed a generic, reproducible Snakemake ^13^ workflow, which conducts all steps from downloading benchmark data, preprocessing, comparison with a known ground truth, plotting, and automatic deployment of the required software stacks via Snakemake’s Conda/Mamba [ 9] integration: https://github.com/snakemake-workflows/dna-seq-benchmark. The workflow comes with predefined standard datasets like CHM-eval and GIAB, but can be additionally configured to use any other DNA-seq-based benchmark dataset consisting of a known set of true variants, confident regions where the reported true variants are considered to be complete ( *i.e.* every non-variant position is assumed to homozygously have the reference allele), raw read data (as FASTQ files), and (optionally) sequenced target regions ( *e.g.* in case of exome sequencing). The workflow uses BWA-mem, ^14^ Picard tools [ 10] and Mosdepth ^15^ for calculating the read coverage across the genome. We use Bedtools ^16^ to limit the known true variants to the confident regions provided by the respective truth publishers and to stratify variants by coverage (see below). For interactive exploration of the results, we use Datavzrd [ 11] and Vega-Lite. ^17^ The matching of calls and true variants in a haplotype-aware manner happens via RTG-tools vcfeval [ 12]. To ensure a fair and correct comparison of the different evaluated callsets, several key points had to be considered, which we outline below.

**Read depth stratification and selection of regions of interest.** The available read depth can naturally affect both the precision and recall of a pipeline. Hence, the read depth characteristics of a benchmark dataset can have an impact on the derived precision and recall, which can limit the generalizability of obtained results. In order to avoid this effect, we decided to stratify recall and precision by read depth. For any benchmark dataset, this workflow generates a quantized set of regions with low (0-9), medium (10-30), and high ( >30 ) read depth using Mosdepth, while considering only reads with mapping quality (MAPQ) ≥60 . Notably, this means that, for example, regions in the low read depth category have either only few reads or a lot of reads with uncertain alignments ( *high mapping uncertainty*). We intersect these regions with the confidence regions of the benchmark sample ( *e.g.* as provided by GIAB) using Bedtools. If the given dataset was generated using a capturing approach ( *e.g.* exome sequencing) we further restrict the regions to the captured loci according to the manufacturer. Afterwards, any given callset is split into three subsets with low, medium, and high coverage using Bedtools.

**Separating genotyping from calling performance** At decreasing read depth or increasing mapping uncertainty, one can expect a callset to yield a decreasing recall: with less evidence, it will become harder to find variants. This is true for both genotyping ( *i.e.* requiring that the variant caller detects the correct genotype) as well as when just requiring the variant allele to be correctly recognized without considering whether the variant is predicted to be homo- or heterozygous ( *i.e.* plain variant *calling* without genotyping). In contrast, a variant callset’s precision should ideally remain constant and unaffected by a decrease in read depth or increase in mapping uncertainty, if the method manages to correctly report the increasing uncertainty with decreasing depth or increasing mapping uncertainty. The latter behavior differs between measuring a callset’s genotyping or calling precision. In order to make these differences visible, we therefore decided to calculate precision and recall for both genotyping and calling separately.

**Variant atomization** Some variant callers report complex variants as replacements of longer alleles ( *i.e.*, both the reported reference and the alternative allele are longer than one base, *e.g.* ACCGCGT>ACGCT). While this is in general a good idea ( *e.g.* in order to be able to properly assess the combined impact on proteins), we found this to introduce problems with vcfeval’s internal comparison approach. This resulted in spurious false positives and false negatives in callsets having such variants. Similar to the approach implemented in the hap.py pipeline [ 13], we solved this issue by introducing a normalization step prior to vcfeval into our analysis workflow, which uses Bcftools ^18^ to normalize variants, in a way that indels are moved to their left-most possible location, and complex replacements are split into their atomic components— *i.e.* single nucleotide variants (SNVs), insertions or deletions (indels)—while removing exact duplicates resulting from the atomization.

**Reporting** For reporting results, we employ Datavzrd to create interactive tabular reports for recall and precision, as well as individual false positive and false negative variants. Datavzrd enables us to just provide the required data as TSV or CSV files combined with a configuration file that defines the rendering of each column. For the latter, one can choose from automatic link-outs, heatmap plots, tick plots, bar plots, or custom complex Vega-Lite plots (which can also be used to define alternative visualizations for an entire table view). For the former, we report a table containing for each callset and each read depth category (low, medium, high) precision and recall (while ignoring whether the genotype was predicted correctly), the underlying counts of true positives (TP), false positives (FP), and false negatives (FN), as well as the fraction of wrongly predicted genotypes. It is important to note that it cannot be excluded that the same variant in the truthset is predicted multiple times by a callset, *e.g.* as part of several complex replacements (see “Variant atomization” above). We therefore report two TP counts ${\mathrm{TP}}_{\text{query}}$ (number of TPs in the callset with the same matching variant from the truth potentially counted multiple times) and ${\mathrm{TP}}_{\text{truth}}$ (number of variants in the truth set that occur in the callset, each variant counted once, regardless how often it occurs in the callset), with ${\mathrm{TP}}_{\text{query}}\geq {\mathrm{TP}}_{\text{truth}}$ . Following the established definitions, precision is then calculated as

$$\frac{{\mathrm{TP}}_{\text{query}}}{{\mathrm{TP}}_{\text{query}}+\mathrm{FP}}$$

while recall is calculated as

$$\frac{{\mathrm{TP}}_{\text{truth}}}{{\mathrm{TP}}_{\text{truth}}+\mathrm{FN}}.$$

An example can be seen in Figure 2. For reporting of individual FP and FN variants, we provide a Datavzrd table view for each that has one row per variant and a column for each callset. In order to visualize systematic patterns arising from the properties of callsets ( *e.g.*, using the same variant detection or mapping method), any kind of property can be annotated as a so-called “label” when registering a callset for evaluation with the pipeline. The labels are displayed using a categorical color coding in the header of the table views. Moreover, we perform a ${\mathrm{Chi}}^{2}$ test for the association of the FP or FN pattern of each variant against the different labels in order to detect systematic effects. The variant/label combinations for which this test yields a significant result are then displayed in a separate table view for each type of label. These allow, for example, to spot variants that only occur when callsets use a particular variant caller. Thereby, significance is determined by controlling the false discovery rate over the p-values of the ${\mathrm{Chi}}^{2}$ test using the Benjamini-Yekuteli procedure, as the variants could be both positively ( *e.g.*, being on the same haplotype) or negatively ( *e.g.*, being on different haplotypes) correlated. In order to combine the results with data provenance information we include the Datavzrd views into a Snakemake report [ 14], which automatically provides a menu structure for navigation between views, association with used parameters, code, and software versions as well as runtime statistics.

### Continuous public evaluation

**Figure 1.**
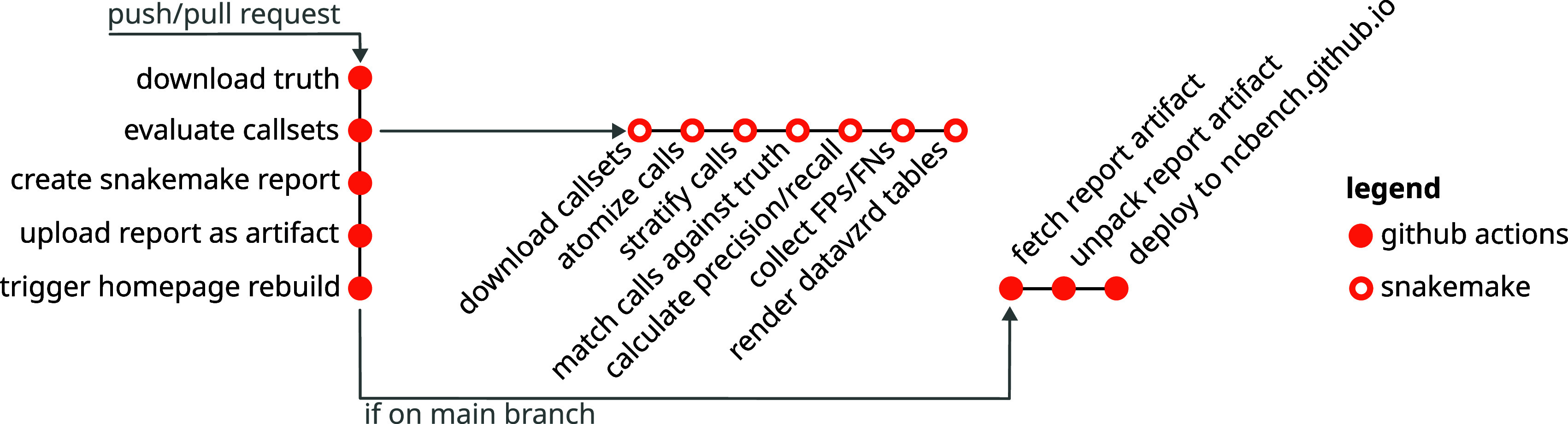
Continuous evaluation and reporting workflow. Upon pull requests or pushes, a GitHub Actions workflow is triggered. This downloads data, runs the Snakemake-based evaluation pipeline, creates the Snakemake report and uploads it as an artifact. If the workflow is triggered on the main branch, its finalization triggers a second Github Actions workflow that builds and deploys the homepage at https://ncbench.github.io.

A central goal of the project was not to conduct a single benchmark and just publish the results, but rather provide a resource for continuous repeated and always up-to-date benchmarking, that is moreover open to any kind of contribution (callsets and code improvements, among others) from outside collaborators. In order to achieve this, we have developed the following approach (see Figure 1 for an illustration). We deployed the benchmarking workflow [ 15] as a module [ 16] into another Snakemake workflow that in addition has the ability to download callsets from Zenodo, using Snakemake’s Zenodo integration [ 17]. Then, we deployed this workflow into the GitHub repository [ 18] and configured GitHub Actions [ 19] to continuously rerun the workflow upon every commit on the main branch or any pull request [ 20]. In order to ensure that the workflow runs sufficiently fast (GitHub Actions offers only limited runtime and resources per job), we have precomputed benchmark dataset-specific central intermediate results (read depth and confidence derived stratification regions) that are computationally intensive to obtain, and deployed them along with the workflow code into the GitHub repository.

Upon each completion of the evaluation pipeline, a Snakemake report [ 21] is generated. In case of pull requests ( *e.g.*, contributing a feature or a new callset), the report is uploaded as a GitHub artifact [ 22], for inspection by the pull request author and the reviewer. In the case of the main branch, we utilize GitHub Actions to trigger the execution of a secondary GitHub Action pipeline in a repository that hosts the NCBench homepage [ 23]. This pipeline fetches the latest report artifact associated with the main branch and deploys it to the homepage. This way, the most recent results are automatically accessible on the homepage.

## Results

The always up-to-date results of the benchmark can be found and interactively explored under https://ncbench.github.io. At the time of writing, the benchmark consists of more than 40 callsets on three different benchmark datasets, the two NA12878 samples described in the Datasets section and CHM-eval. ^19^ The callsets span various pipelines, read mapping, variant detection, and genotyping approaches.

Since the central idea of this project is to provide a continuous, standardized and open benchmark platform for DNA-seq, we strived to make the contribution of new callsets as straightforward as possible. The benchmark repository [ 24] shows the steps needed to perform variant calling on the supported datasets and describes how to pre-check the resulting callset locally. Once a contributor is convinced that the callset is ready for publication, we provide instructions for uploading the result to Zenodo and providing it via a pull request for continuous evaluation in the future.

**Figure 2.**
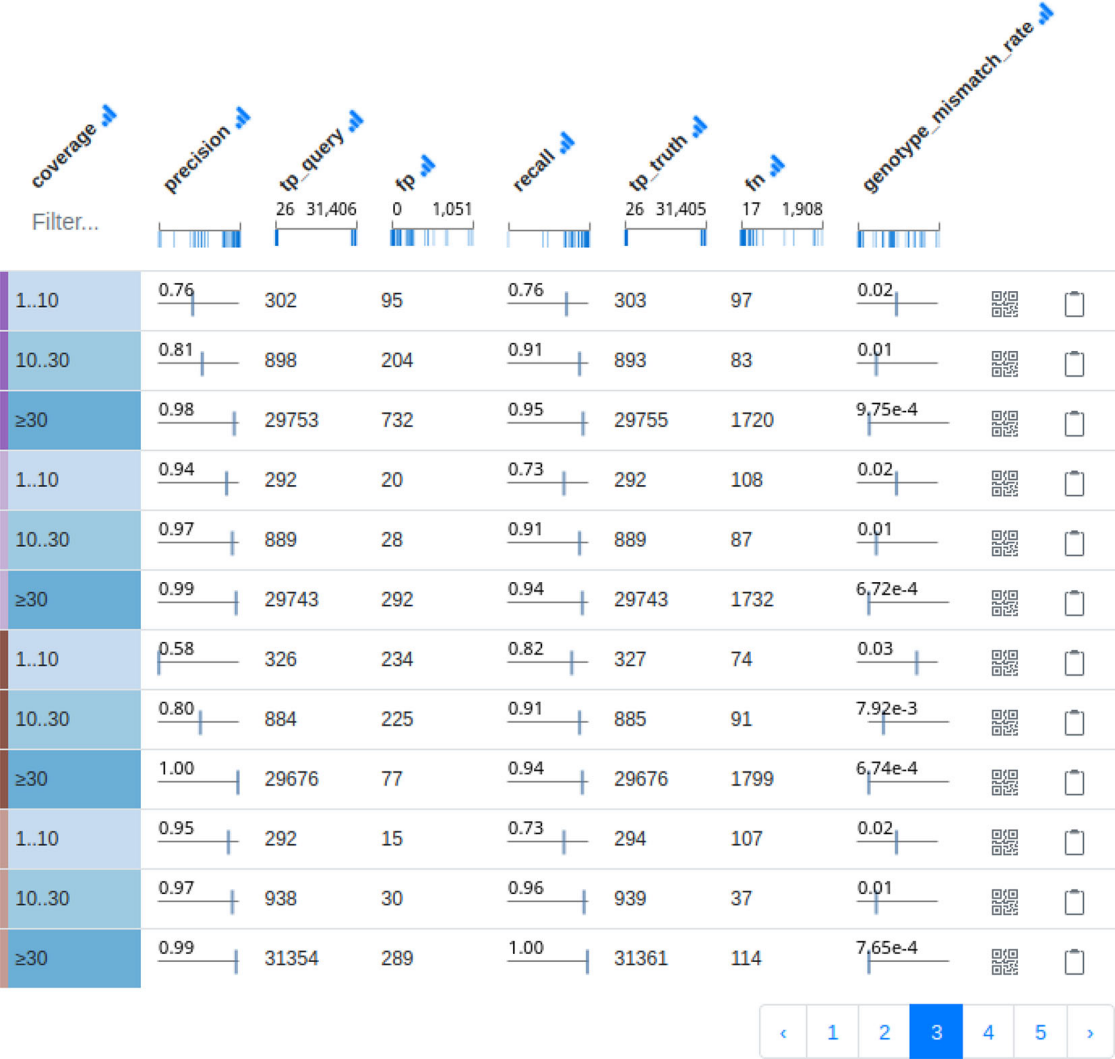
Exemplary screenshot of interactive tabular precision recall display. Each three rows display precision and recall together with underlying numbers and wrongly predicted genotypes stratified by read depth/coverage category. In the interactive report, callset/pipeline names would occur on the left. Here, they have been removed since results can be expected to change over time. For actual results please see the always up-to-date interactive report at https://ncbench.github.io.

Figure 2 shows an exemplary screenshot of the interactive tabular precision/recall display (see Pipeline section). This illustrates the importance of stratifying by read depth/coverage categories (see Pipeline). This is in contrast to the commonly seen practice, where GIAB and other benchmark datasets are evaluated on the entire set of variants, without stratification. While this generates realistic estimates for the prediction quality of a variant calling pipeline overall, the provided information is less generalizable, since a new dataset might have different read depth characteristics. Further, it tells little about the expected quality at an individual location, which might differ as well from the global characteristics.

## Discussion and conclusions

So far, variant calling benchmark studies were often published once, in a single or multiple manuscripts that can only represent a snapshot at the time of writing. This holds both for studies evaluating multiple tools or pipelines, as well as the evaluations around newly published individual tools.

For continuous benchmarking, platforms like Omnibenchmark [ 25] or OpenEBench ^20^ are available. Both platforms run on their own dedicated computing infrastructure and utilize specialized frameworks for results reporting and dataset uploading.

In this work, we demonstrate that a continuous benchmarking platform can be set up without the need for dedicated computing infrastructure, and instead entirely relying on freely available and widely used resources.

• By basing the benchmark of DNA-seq variant calling pipelines on a public GitHub repository for code, configuration and result storage, GitHub Actions for analysis execution, and callsets hosted by Zenodo, we allow rapid and straightforward contributions by anybody used to these services.
• By implementing the analysis with Snakemake and Conda/Mamba, we decouple the analysis code and the reporting of results from the hosting platform: instead of relying on GitHub Actions, the benchmark analysis can easily be conducted locally, or on a different platform without any modifications of the code.
• By generating interactive visual presentations of the results with Datavzrd, we (a) allow for a modern and versatile exploration of results and comparisons between different methods and pipelines, and (b) to a large degree enable contributions and modifications to the way the data is presented by simply editing YAML based configuration files.
• By encapsulating all results in a Snakemake report that is portable and can be viewed and provided without any web service, we enable people to freely choose between relying on the online version of the report and providing snapshot-like versions of the report in their publications.

In the future, we will further extend upon this approach. For example, we will add a whole genome dataset of the NA12878 sample sequenced on an Illumina NovaSeq 6000 of *ca.* 400 million paired-end reads (mean insert size 473 and 2 × 151 bp reads length). Further, we will extend the pipeline to include the evaluation of structural and somatic variants and corresponding datasets. Finally, as the implemented comparison workflow is in principle agnostic to the considered species, we will evaluate the inclusion of benchmark datasets from non-human organisms. Particularly for natural microbial populations, whose species mostly exist as multiple genotypes in one ecosystem, variant calling can be a complex process ^21^ and often not completely resolved due to the lack of complete and closed reference genomes from mono-cultures.

We hope that our approach will attract contributors beyond our initiative. Ideally, the combination of being continuous, simple to use, reproducible, and easy to integrate outside of the primary web service will change the way DNA-seq benchmarking is handled in the future. Instead of requiring every new tool and benchmark study manuscript to conduct its own analysis for precision and recall on public resources like GIAB or CHM-eval as well as comparison with other tools or pipelines, authors can rather include their callsets in our benchmark. In turn, readers will be able to always see the performance of a tool in the context of the state of the art at the time of reading, instead of at the time of writing.

## Author contributions

FH, GG, SM, and JK have written the manuscript. JK has implemented the benchmarking pipeline. SM has coordinated the benchmarking initiative. SM and KB provided the FastQ files for the Agilent Human All Exon v7 kit. MS has created the callset data for the megSAP pipeline and edited the manuscript. TS has created the callset data for the WEScropbio pipeline. LB has created the callset data for the Cologne exome pipeline. AGN analyzed callset data. JA and AO have sequenced the NA12878 sample at the WGGC Cologne. AJP contributed to discussion and manuscript writing. AP has provided advice on and reviewed the benchmark design. SS has created the call sets for the NVIDIA Parabricks pipeline. AB and FB have supported the NVIDIA Parabricks pipeline. AS has coordinated the sequencing of NA12878 at the NGS Core Facility Bonn. NHS and CM have created the callset data for the GHGA pipeline. GG and FH have created the callset data for the sarek pipeline. All authors have read and approved the manuscript.

## Data Availability

Twist Whole-Exome Sequencing Dataset of NA12878: https://doi.org/10.5281/zenodo.7075040 Agilent v7 exomes of NA12878: https://doi.org/10.5281/zenodo.6513788 Data are available under the terms of the Creative Commons Attribution 4.0 International license (CC-BY 4.0). CHM-eval public benchmark data: https://github.com/lh3/CHM-eval NCBench code available from: https://github.com/ncbench/ncbench-workflow Archived NCBench code available from: https://doi.org/10.5281/zenodo.8268264

## Footnotes

^1^

https://openebench.bsc.es

^2^

https://omnibenchmark.org

^3^

https://ngs-kn.de/

^4^

https://www.ghga.de

^5^

https://github.com

^6^

https://github.com/features/actions

^7^

https://zenodo.org

^8^

https://www.nist.gov/programs-projects/genome-bottle

^9^

https://github.com/mamba-org/mamba

^10^

https://broadinstitute.github.io/picard

^11^

https://github.com/datavzrd/datavzrd

^12^

https://github.com/RealTimeGenomics/rtg-tools

^13^

https://github.com/Illumina/hap.py

^14^

https://snakemake.readthedocs.io/en/stable/snakefiles/reporting.html

^15^

https://github.com/snakemake-workflows/dna-seq-benchmark

^16^

https://snakemake.readthedocs.io/en/stable/snakefiles/modularization.html#snakefiles-modules

^17^

https://snakemake.readthedocs.io/en/stable/snakefiles/remote_files.html#zenodo

^18^

https://github.com/ncbench/ncbench-workflow

^19^

https://github.com/features/actions

^20^

https://docs.github.com/en/pull-requests/collaborating-with-pull-requests

^21^

https://snakemake.readthedocs.io/en/stable/snakefiles/reporting.html

^22^

https://docs.github.com/en/actions/using-workflows/storing-workflow-data-as-artifacts

^23^

https://ncbench.github.io

^24^

https://github.com/ncbench/ncbench-workflow

^25^

https://omnibenchmark.org
