# Supplementary material for: NCBench: providing an open, reproducible, transparent, adaptable, and continuous benchmark approach for DNA-sequencing-based variant calling
Source: F1000Res. 2024 Sep 12;12:1125. Originally published 2023 Sep 11. [Version 2] doi: 10.12688/f1000research.140344.2 (PMC11428021; doi:10.12688/f1000research.140344.2)
Supplement: Peer review history

# Reviewer report: Reviewer response for version 2

**DOI:** 10.5256/f1000research.171050.r323086

**Published:** 2024-09-25

**Authors:** Justin M. Zook

**Affiliations:** 1 Material Measurement Laboratory, National Institute of Standards and Technology (NIST), Gaithersburg, MD, USA

The authors have done a good job of addressing the reviewers' questions, and I have no further concerns.

Is the work clearly and accurately presented and does it cite the current literature?

Yes

If applicable, is the statistical analysis and its interpretation appropriate?

Yes

Are all the source data underlying the results available to ensure full reproducibility?

Partly

Is the study design appropriate and is the work technically sound?

Yes

Are the conclusions drawn adequately supported by the results?

Yes

Are sufficient details of methods and analysis provided to allow replication by others?

Partly

Reviewer Expertise:

Variant calling and benchmarking

I confirm that I have read this submission and believe that I have an appropriate level of expertise to confirm that it is of an acceptable scientific standard.

# Reviewer report: Reviewer response for version 2

**DOI:** 10.5256/f1000research.171050.r323085

**Published:** 2024-09-13

**Authors:** Kez Cleal

**Affiliations:** 1 Cardiff University, Cardiff, UK

Thank you for addressing my points in a thorough manner.

Is the work clearly and accurately presented and does it cite the current literature?

Yes

If applicable, is the statistical analysis and its interpretation appropriate?

Not applicable

Are all the source data underlying the results available to ensure full reproducibility?

Yes

Is the study design appropriate and is the work technically sound?

Yes

Are the conclusions drawn adequately supported by the results?

Yes

Are sufficient details of methods and analysis provided to allow replication by others?

Yes

Reviewer Expertise:

Cancer genomics

I confirm that I have read this submission and believe that I have an appropriate level of expertise to confirm that it is of an acceptable scientific standard.

# Reviewer report: Reviewer response for version 1

**DOI:** 10.5256/f1000research.153686.r239648

**Published:** 2024-02-28

**Authors:** Kez Cleal

**Affiliations:** 1 Cardiff University, Cardiff, UK

The article presents NCBench, a benchmarking platform for evaluating SNVs and indel variant calls from various gold-standard benchmark sets. NCBench makes use of public, free infrastructure like GitHub Actions and Zenodo to facilitate continuous, open-source benchmarking, which is a neat idea. NCBench supports evaluation across several datasets, emphasizing adaptability and reproducibility, and effectively addresses the need for ongoing evaluation in genomic research. However, the true utility of NCBench will depend on its adoption by the wider research community which itself could be enhanced by making the platform as easy to use as possible. On this front, I think NCBench could be improved by addressing the following:

  

1. The documentation of how to implement/run NCBench and how to contribute a new callset was pretty limited. A more detailed guide aimed at newcomers, detailing the pipeline steps, inputs and outputs, and ways to configure NCBench for other datasets would help with adoption.
2. It is unclear how to run NCBench locally on a custom benchmark dataset. The pipeline supports uploading a vcf to zenodo and running via github actions. Ideally, NCBench should be possible to run locally to test different tool parameters, or for developers to experiment with new tool implementations. This pattern would make NCBench more useful for general bioinformatics workflows. If this pattern is supported, it should be documented more clearly.
3. The output tables on github.io are useful, but I think they could be improved. For example, it would be useful if rows could be sorted by a column, ability to hide certain columns.
4. There doesn’t appear to be a way to download the results of the benchmarking run, or if there is, I didn’t find it. It would be nice to be able to download results in a table, in order to make custom tables for publication, for example.
5. Stratifying benchmark results by coverage categories is a nice idea. However, I think it would still be useful to include an ‘any’ category (any mapq), to provide a dataset summary. Additionally, if there was a way to filter some of the rows, this would be very useful, for example selecting only mapq 1-10 category.
6. Tables should probably include an F1 score, this would be useful to rank callsets, although which metric used (tp_query or tp_truth) should be evident.
7. The dark-blue colour of some of the cells on the tables makes the text very hard to read (see ‘Show as plot’ subpage).
8. Some of the table numbers needs to be rounded, for example clicking on ‘Show as plot’, the numbers are rounded to >12dp.
9. In some of the tables, the text doesn’t fit on the page (fn variant page, the top row of labels stretched off the page using Safari).

Is the work clearly and accurately presented and does it cite the current literature?

Yes

If applicable, is the statistical analysis and its interpretation appropriate?

Not applicable

Are all the source data underlying the results available to ensure full reproducibility?

Yes

Is the study design appropriate and is the work technically sound?

Yes

Are the conclusions drawn adequately supported by the results?

Yes

Are sufficient details of methods and analysis provided to allow replication by others?

Yes

Reviewer Expertise:

Cancer genomics

I confirm that I have read this submission and believe that I have an appropriate level of expertise to confirm that it is of an acceptable scientific standard, however I have significant reservations, as outlined above.

# Response

**Published:** 2024-08-26

**Authors:** Johannes Köster

**Affiliations:** University of Duisburg-Essen, Germany

We thank the reviewer for the thorough assessment and positive feedback. A point by point response can be found below.

 1. We have added a more detailed documentation on what is required to add new callsets and configure the pipeline to the readme: https://github.com/ncbench/ncbench-workflow

 2. Indeed, we agree that running the workflow locally makes a lot of sense. We have extended the instructions in the readme accordingly.

 3. This is a good suggestion. We have extended Datavzrd accordingly, such that the updated report now supports this functionality, as can be seen in the updated version under https://ncbench.github.io.

 4. Indeed, offering a download is a good suggestion. We have now activated the corresponding feature in datavzrd, such that each table can be downloaded by opening the "hamburger" menu at the top right and clicking the "download as excel" entry.

 5. Filtering of the rows (e.g. by coverage) has already been possible, using the magnifying glass icon on top of the respective column. We explicitly decided against providing a non-stratified precision and recall, because we believe that this is usually misleading, since it does only reflect the composition of easy and harder to call sites in the benchmark sample, rather than the actual performance of the evaluated pipelines/tools. Therefore, any reported such measures would be of very limited generalizability.

 6. We appreciate the suggestion. Instead of F1-score, we have now added the F*-measure, which provides an monotone transformation of F1 that adds additional interpretability, by being an estimate of the probability that a random variant taken from the union of prediction and truth is predicted correctly. We have extended the "Reporting" section in the manuscript accordingly.

 7. We have updated the reports to a newer version of Datavzrd, in which the "show as plot" functionality has been replaced by a functionality that prints the current page as an SVG file that looks exactly the same as the display in the browser, thereby avoiding the low-contrast issue in the previous version.

 8. See answer for point 7, the newer Datavzrd solves this issue.

 9. Indeed, the FN and FP tables are sometimes wider than a usual screen resolution. However, they are scrollable horizontally. We have extended Datavzrd to show a visual indicator that points the user to the scrollbar if that is the case.

# Reviewer report: Reviewer response for version 1

**DOI:** 10.5256/f1000research.153686.r207163

**Published:** 2023-10-03

**Authors:** Justin M. Zook

**Affiliations:** 1 Material Measurement Laboratory, National Institute of Standards and Technology (NIST), Gaithersburg, MD, USA

The authors present an open benchmarking platform for variant calling, with exome and genome variant call sets for two different benchmarks as examples. As the authors note, the ability to continuously benchmark variant calls is currently lacking, as existing efforts like precisionFDA Truth Challenges only reflect a point in time. Ongoing benchmarking should be very useful as both variant calling methods and benchmark sets evolve. I only have a few suggestions for clarifying the data and methods used.

1. Are FNs and FPs counted to include genotype errors, as is the default in vcfeval and hap.py, or do both exclude genotype errors from their counts, which is implied but not explicit in the text
2. I saw the bed file for Agilent but not for twist in zenodo
3. What were the benchmark versions used for GIAB and CHM-eval?
4. Do the authors expect that adding new versions of the benchmarks would be straightforward as GIAB develops these?
5. vcfeval generally should be able to compare different representations of the same variant, as long as they exactly match, even if they are not atomized. The only reason I have found this not to work is if the variant caller gets part of a haplotype wrong or the genotype wrong, in which case the whole haplotype is called wrong even if most of the variants in the region are correct. Is this what the authors’ encountered? If so, it might be good to clarify this. A potential problem with left shifting variants is that occasionally this will cause a change in the haplotype, e.g., if an indel in a homopolymer is shifted past a SNV on the same haplotype, though is relatively rare.
6. The number of variants for CHM-eval is lower than I'd expect. Did the authors restrict to one chromosome?

Is the work clearly and accurately presented and does it cite the current literature?

Yes

If applicable, is the statistical analysis and its interpretation appropriate?

Yes

Are all the source data underlying the results available to ensure full reproducibility?

Partly

Is the study design appropriate and is the work technically sound?

Yes

Are the conclusions drawn adequately supported by the results?

Yes

Are sufficient details of methods and analysis provided to allow replication by others?

Partly

Reviewer Expertise:

Variant calling and benchmarking

I confirm that I have read this submission and believe that I have an appropriate level of expertise to confirm that it is of an acceptable scientific standard, however I have significant reservations, as outlined above.

# Response

**Published:** 2024-08-26

**Authors:** Johannes Köster

**Affiliations:** University of Duisburg-Essen, Germany

We thank the reviewer for the positive and comprehensive assessment of our work. Please find a point by point response below.

 1. TP, FP, and FN are calculated by ignoring the genotype (just requiring presence or absense of the variant in the callset). Instead, we decided to provide a genotype mismatch rate, that shows how many of the genotypes in the true positives are wrongly predicted compared to the truth. The definition of this approach can be found under "Methods"/"Evaluation pipleine"/"Reporting".

 2. The twist bed file can be found publicly under https://www.twistbioscience.com/sites/default/files/resources/2022-01/Twist_Comprehensive_Exome_Covered_Targets_hg38.bed. This is also the file that is used by the ncbench workflow when assessing the twist dataset. The user does not need the bedfile, since ncbench takes care of restricting any input vcf to calls within the regions specified by the bed file.

 3. The pipeline currently uses v4.2.1 of the NA12878 ground truth from GIAB and v0.5 of CHM-eval. We have extended the online report to include this information in the description above each result table.

 4. Indeed, bumping benchmark versions is very easy. One simply has to update the respective entries here: https://github.com/snakemake-workflows/dna-seq-benchmark/blob/main/workflow/resources/presets.yaml#L51. We have added a section about the maintenance to the end of the "Evaluation pipeline" section in the manuscript.

 5. We have extended and clarified our statement (see section "variant atomization"). Indeed, it is like suggested: the whole replacement is considered as one, leading to missing additional true positives within the replacement.

 6. There was no restriction to a particular chromosome involved. To be sure, we have checked the number of variants in the CHM-eval ground truth and can confirm that it matches the reported TP and FN in the reported tables. Note that (a) the precision/recall tables are split into SNVs and indels, such that both numbers have to be considered. (b) we filter the truth to the reported confidence regions. (c) we stratify by coverage in a way that each kept variant has to be entirely inside of the respective coverage region. In total, this explains that the benchmark runs on fewer variants than one would consider otherwise.
